# Supplementary material for: Neural Correlates of Anesthesia in Newborn Mice and Humans
Source: Front Neural Circuits. 2019 May 22;13:38. doi: 10.3389/fncir.2019.00038 (PMC6538977; doi:10.3389/fncir.2019.00038)
Supplement: Supplementary file 1 [file Data_Sheet_1.pdf]

Supplementary Materials

Neural correlates of anesthesia in newborn mice and humans

Mattia Chini[1], Sabine Gretenkord[1], Johanna K. Kostka[1], Jastyn A. Pöpplau[1], Laura Cornelissen[2,3], Charles B. Berde[2,3], Ileana L. Hanganu-Opatz[1,*] & Sebastian H. Bitzenhofer[1,†*]

[1] Developmental Neurophysiology, Institute of Neuroanatomy, University Medical Center Hamburg-Eppendorf, Hamburg, Germany
[2] Department of Anesthesiology, Critical Care and Pain Medicine, Boston Children's Hospital, Boston, Massachusetts
[3] Department of Anaesthesia, Harvard Medical School, Boston, Massachusetts
[†] Present address: Center for Neural Circuits and Behavior, Department of Neurosciences, University of California, San Diego, La Jolla, CA, USA

* Equal contribution

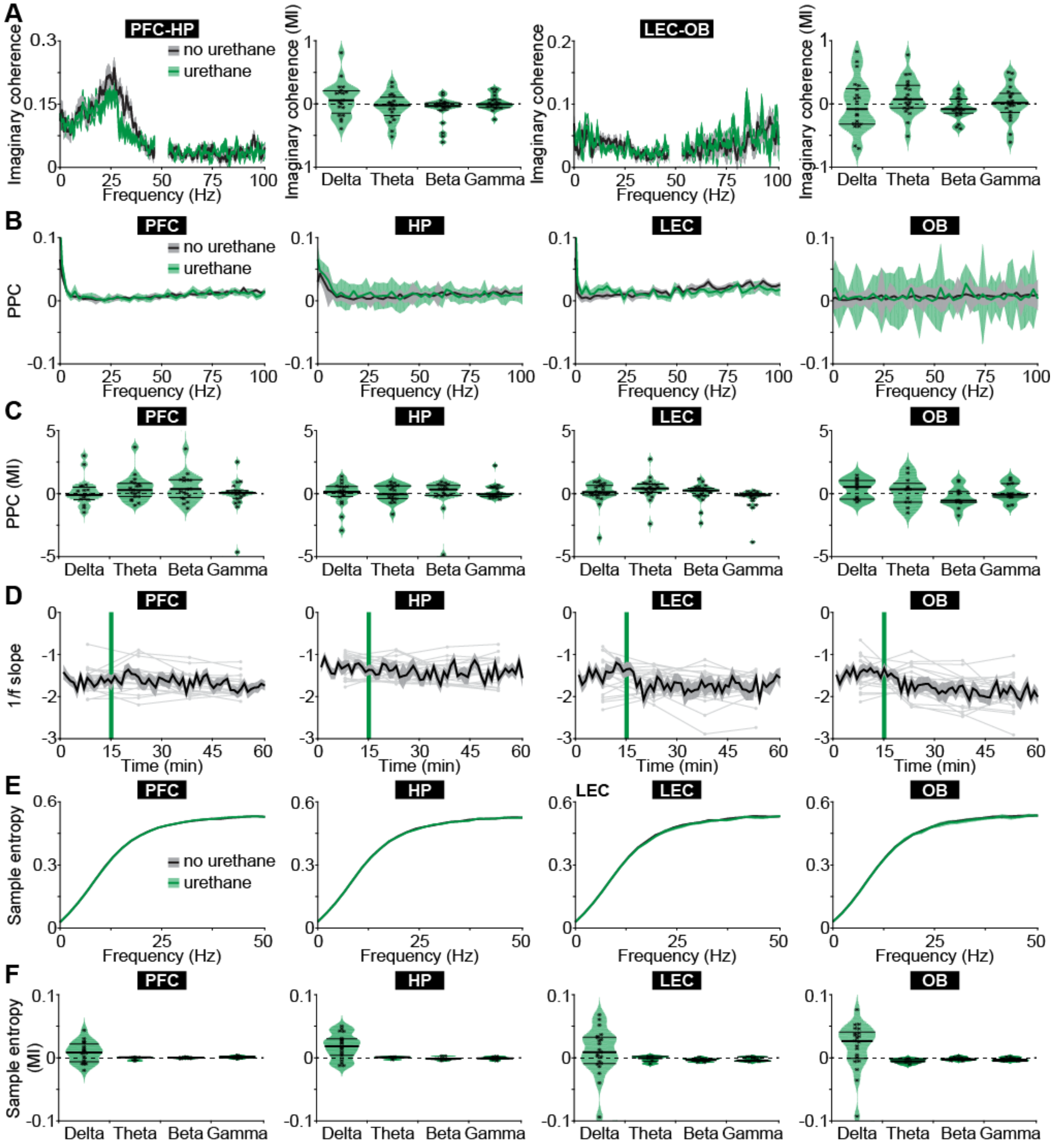

Fig. S1. Urethane anesthesia does not affect spectral features and timing of activity in neonatal mice. (**A**) Line plots displaying the imaginary coherence between PFC-HP and LEC-OB in neonatal mice (P8-10) as a function of frequency before (black) and after (green) urethane injection. Violin plots displaying the MI of the imaginary coherence in delta (2-4 Hz), theta-alpha (4-12 Hz), beta (12-30 Hz) and gamma (30-100 Hz) frequency bands. (**B**) Line plots displaying the PPC of MUA to the oscillatory phase before and after urethane injection. (**C**) Violin plots displaying the MI of PPC in delta, theta, beta and gamma frequency bands. (**D**) Line plots displaying the slope of the 1/f decay for gamma frequencies over time. Green lines mark the time point of urethane injection. (**E**) Line plots displaying the sample entropy as a function of frequency before and after urethane

injection. (**F**) Violin plots displaying the MI of the sample entropy in delta, theta, beta and gamma frequency bands.

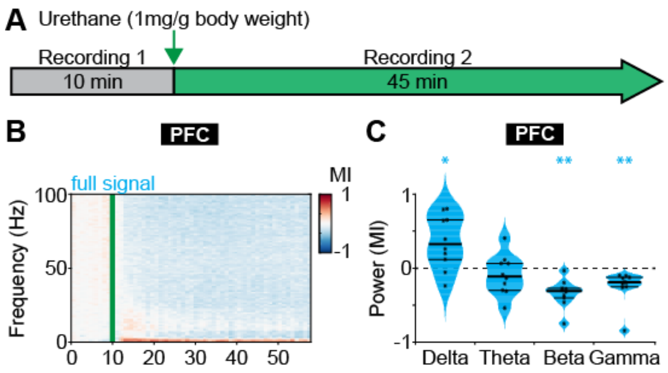

Fig. S2. Frequency-specific effects of urethane anesthesia in juvenile mice. (**A**) Schematic representation of experimental paradigm of LFP recordings in PFC of non-anesthetized and urethane-anesthetized juvenile mice (P24-39). (**B**) Color-coded MI of oscillatory power for full signal before and after urethane injection. Green line corresponds to the time point of urethane injection. (**C**) Violin plots displaying the MI of oscillatory power in delta (2-4 Hz), theta-alpha (4-12 Hz), beta (12-30 Hz) and gamma (30-100 Hz) frequency bands for full signal.

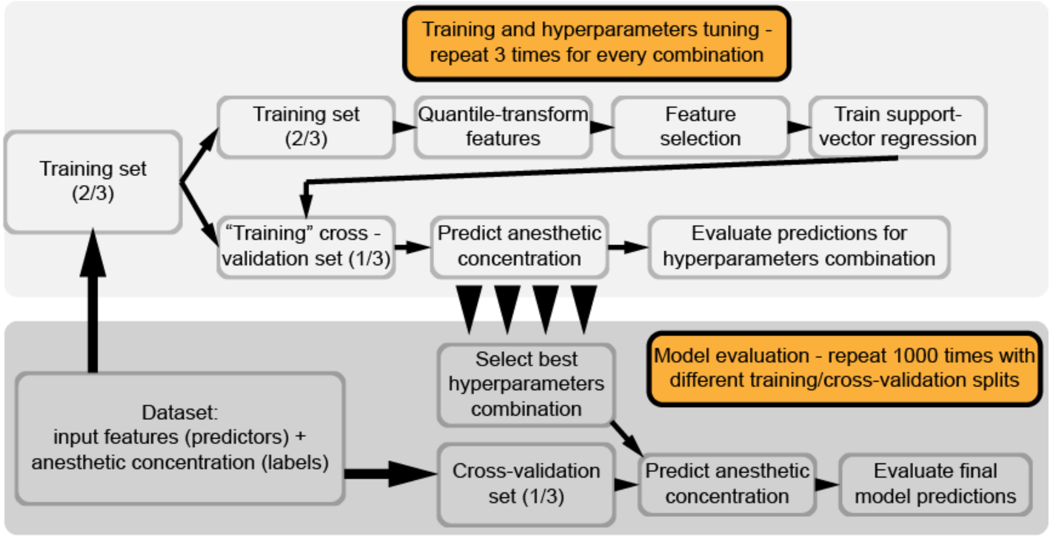

Fig. S3. Machine learning algorithm. Flowchart depicting steps for machine learning algorithm.

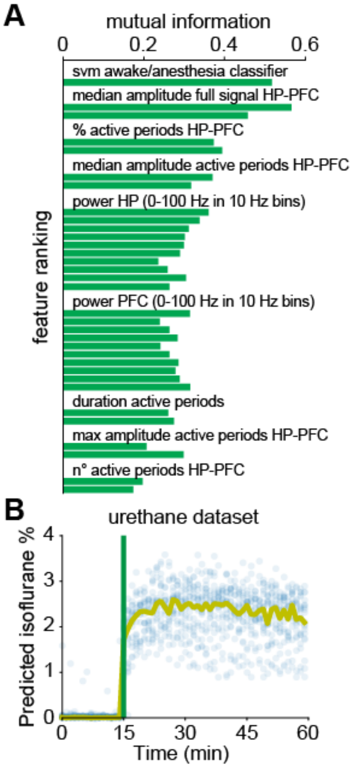

Fig. S4. Median amplitude is most informative for predicting anesthetic concentration in neonatal mice. (**A**) Bar plot displaying the feature ranking for anesthesia depth prediction by mutual information between each feature and anesthesia depth. (**B**) Scatter plot displaying predicted isoflurane concentration using features of LFP recordings from PFC and HP of urethane-anesthetized mice. Green line marks the time point of urethane injection.

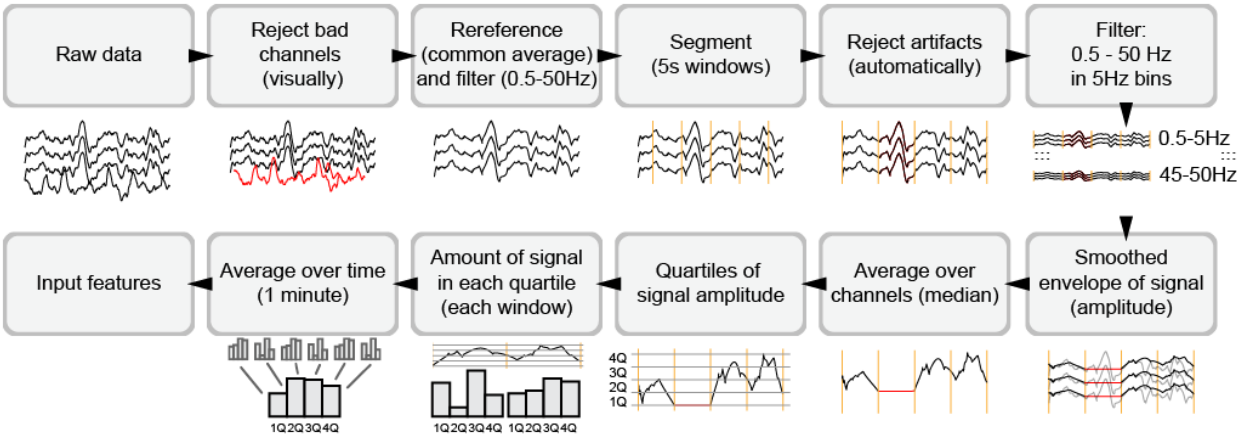

Fig. S5. EEG data processing. Flowchart depicting analysis steps for EEG data processing.

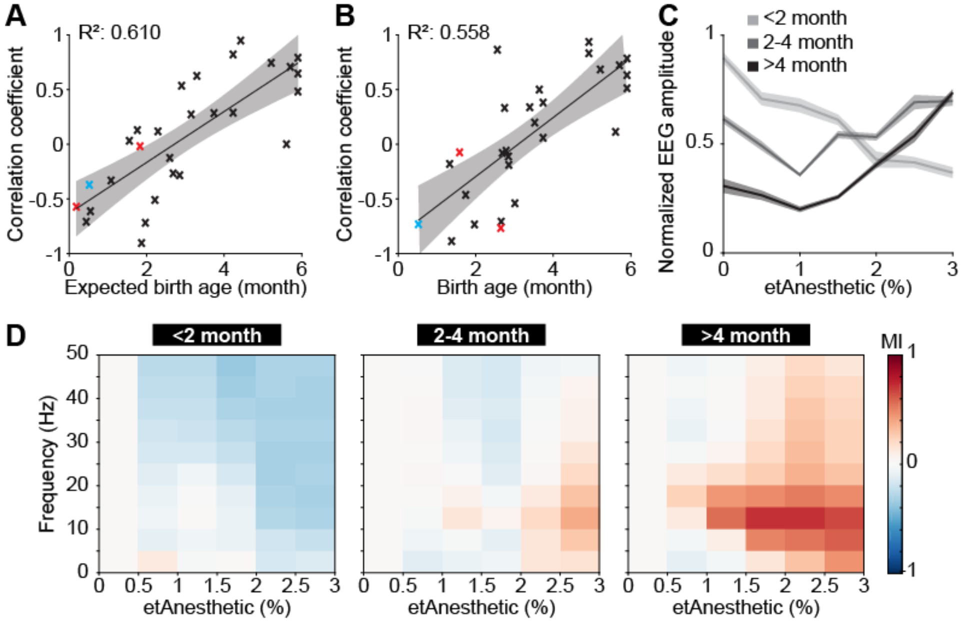

Fig. S6. Age-dependent switch from broadband suppression to frequency-specific effects of general anesthesia on EEG activity for post conceptual age and frontal electrodes. (**A**) Scatter plot displaying the correlation coefficient of median EEG amplitude and anesthetic concentration in relationship to expected birth age for sevoflurane (black), isoflurane (red), and desflurane (blue). (**B**) Scatter plot displaying the correlation coefficient of median EEG amplitude of frontal electrodes and anesthetic concentration in relationship to birth age for sevoflurane (black), isoflurane (red), and desflurane (blue). (**C**) Line plots displaying normalized EEG amplitude of frontal electrodes as a function of anesthetic concentration. (**D**) Color-coded MI of median EEG amplitudes of frontal electrodes in different frequency bands as a function of anesthetic concentration for human babies of 0-2 months (left), 2-4 months (middle) and 4-6 months (right).

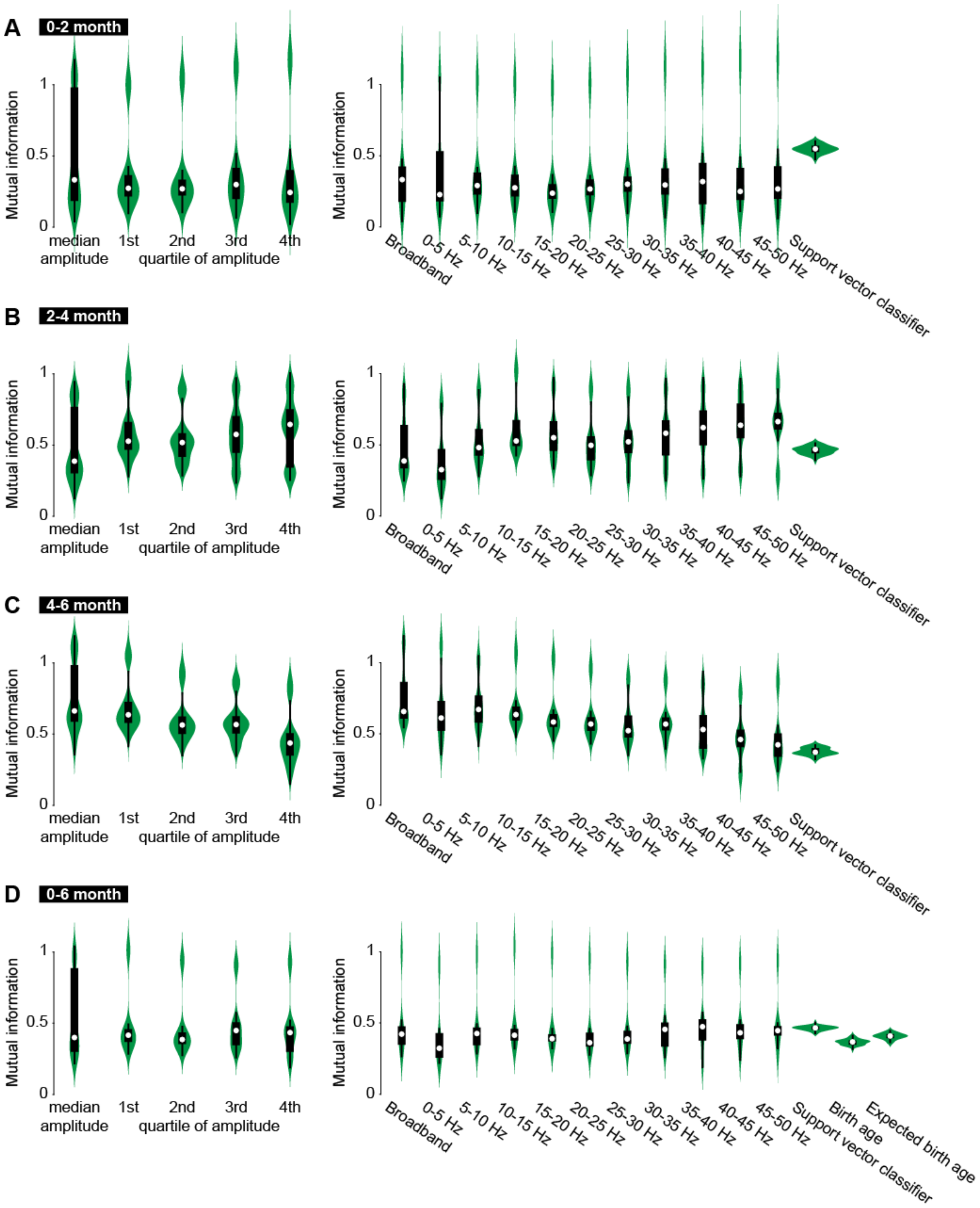

Fig. S7. Features predicting anesthetic concentration from EEG recordings in human infants. (**A**) Violin plots displaying mutual information between each feature and predicted anesthetic concentration for amplitude-related features (left) and frequency-related features (right) for human infants of 0-2 months of age. (**B**) Same as (A) for human

infants of 2-4 months of age. (**C**) Same as (A) for human infants of 4-6 months of age. (**D**) Same as (A) for human infants of 0-6 months of age.

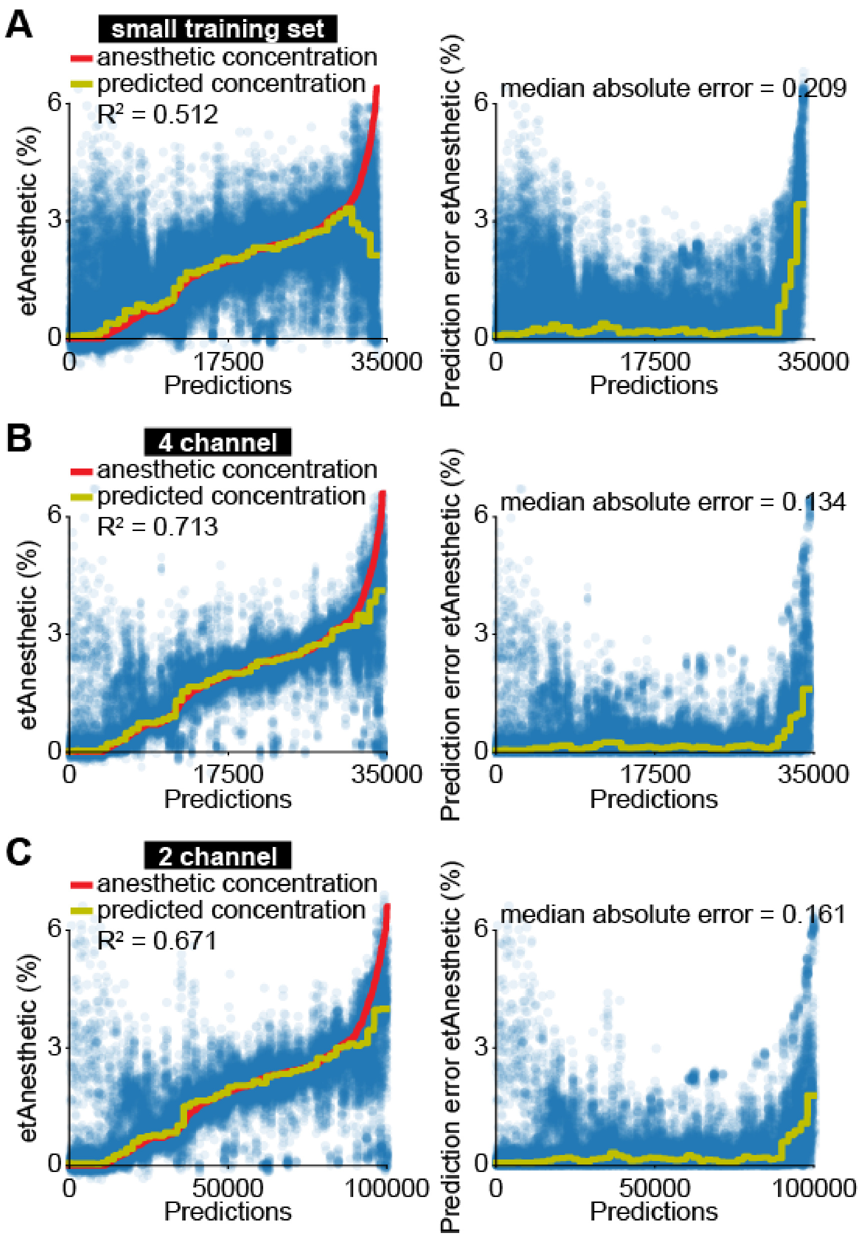

Fig. S8. Reduced data set of EEG activity is still predictive for anesthetic concentration in human infants. (**A**) Scatter plots displaying measured anesthetic concentration and predicted anesthetic concentration of support vector regression for all predictions (left) and absolute difference between measured and predicted anesthetic concentration (right) for a reduced training data set of 20%. (**B**) Scatter plots displaying measured anesthetic concentration and predicted anesthetic concentration of support vector regression for all predictions (left) and absolute difference between measured and predicted anesthetic concentration (right) for reduced EEG data of 4 channels (F4, P4, F3, P3). (**C**) Same as (B) for reduced EEG data of 2 channels (P4, P3).

Tab. S1. Demographic information.

	All Subjects	Age Groups		
	0-6 months	0-2 months	2-4 months	4-6 months
	N=35	n=6	n=19	n=10
Age at birth (weeks; median, IQR)	38.00 [36.64, 39.00]	39.71 [38.25, 40.53]	37.00 [34.00, 39.00]	39.00 [37.44, 39.00]
PNA (months; median, IQR))	3.06 [2.64, 4.42]	1.51 [1.36, 1.73]	2.89 [2.74, 3.52]	5.54 [5.08, 5.91]
Weight (kg, median, IQR)	5.97 [4.89, 7.09]	4.91 [4.80, 5.01]	5.76 [4.89, 6.62]	7.29 [6.42, 8.10]
Male (n, %)	30 (85.7)	5 (83.3)	16 (84.2)	9 (90.0)
Duration of Anesthesia (mins; median, IQR)	114.00 [82.5, 181.00]	190.00 [123.50, 211.50]	114.00 [85.00, 154.00]	89.00 [76.5, 181.75]

ID	Age at birth (weeks)	Postnatal age (months)	Weight (kg)	Sex	Surgery	Duration Anesthesia
1	39,0	0,53	3,7	Female	Anorectoplasty	217
2	38,0	1,35	5,7	Male	Hernia Repair	103
3	41,0	1,41	4,8	Male	Hernia Repair	93
4	40,4	1,61	5,0	Male	Extrophy of Bladder Closure, Spica Cast	185
5	40,6	1,77	4,8	Male	Colostomy Closure	268
6	37,5	1,87	5,0	Male	Hernia Repair, Frenulotomy	195
7	39,0	2,04	5,0	Male	Circumcision	60
8	42,0	2,60	6,0	Male	Hernia Repair	89
9	34,0	2,60	4,8	Male	Hernia Repair	103
10	29.1	2,69	3,3	Male	Hernia Repair, Circumcision	152
11	39,0	2,73	7,2	Male	Hernia Repair, Orchidopexy	187
12	30,3	2,76	3,4	Male	Hernia Repair	149
13	35,4	2,79	4,7	Male	Hernia Repair, Meatoplasty	114
14	34,0	2,83	5,0	Male	Hernia Repair	79
15	39,0	2,89	6,1	Female	Hernia Repair	96
16	37,0	2,89	6,3	Male	Hernia Repair	140
17	39,0	3,02	5,8	Female	Vaginoscopy	156

18	29,0	3,06	4,2	Male	Hernia Repair	185
19	38,0	3,45	6,9	Male	Hernia Repair	72
20	39,0	3,52	7,2	Male	Fistulotomy	58
21	37,0	3,52	6,1	Female	Nephrectomy	170
22	37,0	3,58	5,7	Male	Hernia Repair	118
23	41,0	3,71	5,8	Male	Hernia Repair	102
24	39,0	3,81	7,0	Male	Pyleoplasty	177
25	39,0	3,81	7,6	Male	Fistulotomy	81
26	39,0	4,04	7,4	Male	Hernia Repair	94
27	42,0	4,80	6,3	Female	Hernia Repair	76
28	29,1	5,03	7,8	Male	Hernia Repair	78
29	38,0	5,26	6,3	Male	Hypospadias Repair	22
30	39,0	5,36	8,5	Male	Orchidopexy	76
31	39,1	5,72	7,2	Male	Colostomy Closure	310
32	29,0	5,78	9,1	Male	Fistulotomy	62
33	40,4	5,95	8,2	Male	Hypospadias Repair	189
34	39,0	6,01	6,7	Male	Chordee Release	160
35	30,3	6,05	3,2	Male	Circumcision	84

Tab. S2. Statistics summary.

Figure 1B

Active periods PFC
one-way anova
Analysis of Variance of Aligned Rank Transformed Data

Table Type: Repeated Measures Analysis of Variance Table (Type I)
Model: Repeated Measures (aov)
Response: art(variable)

```
Error Df Df.res F value Pr(>F)
1 time anm:t 3 54 59.792 < 2.22e-16 ***
---
Signif. codes: 0 '***' 0.001 '**' 0.01 '*' 0.05 '.' 0.1 ' ' 1
contrast estimate SE df t.ratio p.value
1 - 2 35.000000 3.280084 54 10.670 <.0001
1 - 3 40.105263 3.280084 54 12.227 <.0001
```

Figure 1B

Active periods HP
one-way anova
Analysis of Variance of Aligned Rank Transformed Data

Table Type: Repeated Measures Analysis of Variance Table (Type I)
Model: Repeated Measures (aov)
Response: art(variable)

```
Error Df Df.res F value Pr(>F)
1 time anm:t 3 54 35.13 9.916e-13 ***
---
Signif. codes: 0 '***' 0.001 '**' 0.01 '*' 0.05 '.' 0.1 ' ' 1
contrast estimate SE df t.ratio p.value
1 - 2 31.894737 3.912163 54 8.153 <.0001
1 - 3 36.631579 3.912163 54 9.364 <.0001
```

Figure 1B

Active periods LEC
one-way anova
Analysis of Variance of Aligned Rank Transformed Data

Table Type: Repeated Measures Analysis of Variance Table (Type I)
Model: Repeated Measures (aov)
Response: art(variable)

```
Error Df Df.res F value Pr(>F)
1 time anm:t 3 60 29.392 8.1185e-12 ***
---
Signif. codes: 0 '***' 0.001 '**' 0.01 '*' 0.05 '.' 0.1 ' ' 1
contrast estimate SE df t.ratio p.value
1 - 2 26.285714 3.86664 60 6.798 <.0001
1 - 3 29.142857 3.86664 60 7.537 <.0001
```

Figure 1B

Active periods OB
one-way anova
Analysis of Variance of Aligned Rank Transformed Data

Table Type: Repeated Measures Analysis of Variance Table (Type I)
Model: Repeated Measures (aov)
Response: art(variable)

```
Error Df Df.res F value Pr(>F)
1 time anm:t 3 60 27.283 2.9636e-11 ***
---
Signif. codes: 0 '***' 0.001 '**' 0.01 '*' 0.05 '.' 0.1 ' ' 1
contrast estimate SE df t.ratio p.value
1 - 2 28.4761905 4.2771 60 6.658 <.0001
1 - 3 32.5714286 4.2771 60 7.615 <.0001
```

1 - 4 29.000000 3.280084 54 8.841 <.0001
2 - 3 5.105263 3.280084 54 1.556 0.4118
2 - 4 -6.000000 3.280084 54 -1.829 0.2711
3 - 4 -11.105263 3.280084 54 -3.386 0.0071

P value adjustment: tukey method for comparing a family of 4 estimates

Figure 1D

Power full signal PFC
two-way anova
Analysis of Variance of Aligned Rank Transformed Data

Table Type: Repeated Measures Analysis of Variance Table (Type I)
Model: Repeated Measures (aov)
Response: art(variable)

Error Df Df.res F value Pr(>F)
1 cond anm:c 1 18 69.487 1.3576e-07 ***
2 cond:freq anm:: 3 54 61.935 < 2.22e-16 ***

Signif. codes: 0 '***' 0.001 '**' 0.01 '*' 0.05 '.' 0.1 ' ' 1

Bonferroni post-hoc comparison
delta theta beta gamma
3.051758e-05 1.525879e-05 1.525879e-05 1.525879e-05

Power active periods PFC
two-way anova
Analysis of Variance of Aligned Rank Transformed Data

Table Type: Repeated Measures Analysis of Variance Table (Type I)
Model: Repeated Measures (aov)
Response: art(variable)

Error Df Df.res F value Pr(>F)
1 cond anm:c 1 18 22.274 0.00017099 ***
2 cond:freq anm:: 3 54 13.800 8.5004e-07 ***

Signif. codes: 0 '***' 0.001 '**' 0.01 '*' 0.05 '.' 0.1 ' ' 1

1 - 4 26.947368 3.912163 54 6.888 <.0001
2 - 3 4.736842 3.912163 54 1.211 0.6228
2 - 4 -4.947368 3.912163 54 -1.265 0.5891
3 - 4 -9.684211 3.912163 54 -2.475 0.0754

P value adjustment: tukey method for comparing a family of 4 estimates

Figure 1D

Power full signal HP
two-way anova
Analysis of Variance of Aligned Rank Transformed Data

Table Type: Repeated Measures Analysis of Variance Table (Type I)
Model: Repeated Measures (aov)
Response: art(variable)

Error Df Df.res F value Pr(>F)
1 cond anm:c 1 18 46.382 2.2384e-06 ***
2 cond:freq anm:: 3 54 48.874 2.0854e-15 ***

Signif. codes: 0 '***' 0.001 '**' 0.01 '*' 0.05 '.' 0.1 ' ' 1

Bonferroni post-hoc comparison
delta theta beta gamma
1.525879e-05 1.525879e-05 1.525879e-05 1.525879e-05

Power active periods HP
two-way anova
Analysis of Variance of Aligned Rank Transformed Data

Table Type: Repeated Measures Analysis of Variance Table (Type I)
Model: Repeated Measures (aov)
Response: art(variable)

Error Df Df.res F value Pr(>F)
1 cond anm:c 1 18 13.4602 0.0017565 **
2 cond:freq anm:: 3 54 4.4602 0.0071739 **

Signif. codes: 0 '***' 0.001 '**' 0.01 '*' 0.05 '.' 0.1 ' ' 1

1 - 4 4.666667 3.86664 60 1.207 0.6249
2 - 3 2.857143 3.86664 60 0.739 0.8810
2 - 4 -21.619048 3.86664 60 -5.591 <.0001
3 - 4 -24.476190 3.86664 60 -6.330 <.0001

P value adjustment: tukey method for comparing a family of 4 estimates

Figure 1D

Power full signal LEC
two-way anova
Analysis of Variance of Aligned Rank Transformed Data

Table Type: Repeated Measures Analysis of Variance Table (Type I)
Model: Repeated Measures (aov)
Response: art(variable)

Error Df Df.res F value Pr(>F)
1 cond anm:c 1 20 29.561 2.5383e-05 ***
2 cond:freq anm:: 3 60 14.028 4.8921e-07 ***

Signif. codes: 0 '***' 0.001 '**' 0.01 '*' 0.05 '.' 0.1 ' ' 1

Bonferroni post-hoc comparison
delta theta beta gamma
0.08628464 4.703522e-03 3.814697e-06 0.0003356934

Power active periods LEC
two-way anova
Analysis of Variance of Aligned Rank Transformed Data

Table Type: Repeated Measures Analysis of Variance Table (Type I)
Model: Repeated Measures (aov)
Response: art(variable)

Error Df Df.res F value Pr(>F)
1 cond anm:c 1 20 17.512 0.00045669 ***
2 cond:freq anm:: 3 60 11.020 7.3369e-06 ***

Signif. codes: 0 '***' 0.001 '**' 0.01 '*' 0.05 '.' 0.1 ' ' 1

1 - 4 32.9523810 4.2771 60 7.704 <.0001
2 - 3 4.0952381 4.2771 60 0.957 0.7739
2 - 4 4.4761905 4.2771 60 1.047 0.7229
3 - 4 0.3809524 4.2771 60 0.089 0.9997

P value adjustment: tukey method for comparing a family of 4 estimates

Figure 1D

Power full signal OB
two-way anova
Analysis of Variance of Aligned Rank Transformed Data

Table Type: Repeated Measures Analysis of Variance Table (Type I)
Model: Repeated Measures (aov)
Response: art(variable)

Error Df Df.res F value Pr(>F)
1 cond anm:c 1 20 4.2525 0.052427 .
2 cond:freq anm:: 3 60 3.6152 0.018139 *

Signif. codes: 0 '***' 0.001 '**' 0.01 '*' 0.05 '.' 0.1 ' ' 1

Bonferroni post-hoc comparison
delta theta beta gamma
1.00000000 2.883911e-03 1.171112e-03 0.0171394348

Power active periods OB
two-way anova
Analysis of Variance of Aligned Rank Transformed Data

Table Type: Repeated Measures Analysis of Variance Table (Type I)
Model: Repeated Measures (aov)
Response: art(variable)

Error Df Df.res F value Pr(>F)
1 cond anm:c 1 20 1.3282 0.262711
2 cond:freq anm:: 3 60 2.5393 0.064904 .

Signif. codes: 0 '***' 0.001 '**' 0.01 '*' 0.05 '.' 0.1 ' ' 1

Bonferroni post-hoc comparison
delta theta beta gamma
2.471924e-02 9.650116e-01
1.000000e+00 2.857971e-02

Figure 1E

Log firing rate full signal PFC
one-way anova
Analysis of Variance of Aligned Rank Transformed Data

Table Type: Repeated Measures Analysis of Variance Table (Type I)
Model: Repeated Measures (aov)
Response: art(variable)

Error Df Df.res F value Pr(>F)
1 time anm:t 3 54 27.752 5.3925e-11 ***

Signif. codes: 0 '***' 0.001 '**' 0.01 '*' 0.05 '.' 0.1 ' ' 1
contrast estimate SE df t.ratio p.value
1 - 2 18.3684211 2.347886 54 7.823 <.0001
1 - 3 18.5263158 2.347886 54 7.891 <.0001
1 - 4 14.1578947 2.347886 54 6.030 <.0001
2 - 3 0.1578947 2.347886 54 0.067 0.9999
2 - 4 -4.2105263 2.347886 54 -1.793 0.2877
3 - 4 -4.3684211 2.347886 54 -1.861 0.2571

P value adjustment: tukey method for comparing a family of 4 estimates

Log firing rate active periods PFC
one-way anova
Analysis of Variance of Aligned Rank Transformed Data

Table Type: Repeated Measures Analysis of Variance Table (Type I)
Model: Repeated Measures (aov)
Response: art(variable)

Error Df Df.res F value Pr(>F)
1 time anm:t 3 54 4.7319 0.0052906 **

Bonferroni post-hoc comparison
delta theta beta gamma
9.012909e-01 7.232666e-02
4.943848e-02 6.256104e-01

Figure 1E

Log firing rate full signal HP
one-way anova
Analysis of Variance of Aligned Rank Transformed Data

Table Type: Repeated Measures Analysis of Variance Table (Type I)
Model: Repeated Measures (aov)
Response: art(variable)

Error Df Df.res F value Pr(>F)
1 time anm:t 3 54 13.869 8.0251e-07 ***

Signif. codes: 0 '***' 0.001 '**' 0.01 '*' 0.05 '.' 0.1 ' ' 1
contrast estimate SE df t.ratio p.value
1 - 2 9.3684211 1.71104 54 5.475 <.0001
1 - 3 9.7105263 1.71104 54 5.675 <.0001
1 - 4 5.8684211 1.71104 54 3.430 0.0062
2 - 3 0.3421053 1.71104 54 0.200 0.9971
2 - 4 -3.5000000 1.71104 54 -2.046 0.1844
3 - 4 -3.8421053 1.71104 54 -2.245 0.1241

P value adjustment: tukey method for comparing a family of 4 estimates

Log firing rate active periods HP
one-way anova
Analysis of Variance of Aligned Rank Transformed Data

Table Type: Repeated Measures Analysis of Variance Table (Type I)
Model: Repeated Measures (aov)
Response: art(variable)

Error Df Df.res F value Pr(>F)
1 time anm:t 3 54 1.9002 0.14053

Bonferroni post-hoc comparison
delta theta beta gamma
1.00000000 1.907349e-05
4.703522e-03 0.0862846375

Figure 1E

Log firing rate full signal LEC
one-way anova
Analysis of Variance of Aligned Rank Transformed Data

Table Type: Repeated Measures Analysis of Variance Table (Type I)
Model: Repeated Measures (aov)
Response: art(variable)

Error Df Df.res F value Pr(>F)
1 time anm:t 3 60 12.998 1.204e-06 ***

Signif. codes: 0 '***' 0.001 '**' 0.01 '*' 0.05 '.' 0.1 ' ' 1
contrast estimate SE df t.ratio p.value
1 - 2 1.123810e+01 2.139859 60 5.252 <.0001
1 - 3 1.123810e+01 2.139859 60 5.252 <.0001
1 - 4 1.009524e+01 2.139859 60 4.718 0.0001
2 - 3 1.421085e-14 2.139859 60 0.000 1.0000
2 - 4 -1.142857e+00 2.139859 60 -0.534 0.9504
3 - 4 -1.142857e+00 2.139859 60 -0.534 0.9504

P value adjustment: tukey method for comparing a family of 4 estimates

Log firing rate active periods LEC
one-way anova
Analysis of Variance of Aligned Rank Transformed Data

Table Type: Repeated Measures Analysis of Variance Table (Type I)
Model: Repeated Measures (aov)
Response: art(variable)

Error Df Df.res F value Pr(>F)
1 time anm:t 3 60 11.826 3.4682e-06 ***

Bonferroni post-hoc comparison
delta theta beta gamma
0.11605835 9.713669e-01
2.840424e-02 0.0862846375

Figure 1E

Log firing rate full signal OB
one-way anova
Analysis of Variance of Aligned Rank Transformed Data

Table Type: Repeated Measures Analysis of Variance Table (Type I)
Model: Repeated Measures (aov)
Response: art(variable)

Error Df Df.res F value Pr(>F)
1 time anm:t 3 60 5.1927 0.0029577 **

Signif. codes: 0 '***' 0.001 '**' 0.01 '*' 0.05 '.' 0.1 ' ' 1
contrast estimate SE df t.ratio p.value
1 - 2 6.3333333 2.434027 60 2.602 0.0551
1 - 3 9.4047619 2.434027 60 3.864 0.0015
1 - 4 5.6904762 2.434027 60 2.338 0.1009
2 - 3 3.0714286 2.434027 60 1.262 0.5905
2 - 4 -0.6428571 2.434027 60 -0.264 0.9935
3 - 4 -3.7142857 2.434027 60 -1.526 0.4286

P value adjustment: tukey method for comparing a family of 4 estimates

Log firing rate active periods OB
one-way anova
Analysis of Variance of Aligned Rank Transformed Data

Table Type: Repeated Measures Analysis of Variance Table (Type I)
Model: Repeated Measures (aov)
Response: art(variable)

Error Df Df.res F value Pr(>F)
1 time anm:t 3 60 1.0235 0.38868

Signif. codes: 0 ‘***’ 0.001 ‘**’ 0.01 ‘*’ 0.05 ‘.’ 0.1 ‘ ’ 1
contrast estimate SE df t.ratio p.value
1 - 2 7.3684211 2.394314 54 3.077 0.0168
1 - 3 7.7368421 2.394314 54 3.231 0.0110
1 - 4 6.8947368 2.394314 54 2.880 0.0283
2 - 3 0.3684211 2.394314 54 0.154 0.9987
2 - 4 -0.4736842 2.394314 54 -0.198 0.9972
3 - 4 -0.8421053 2.394314 54 -0.352 0.9849

P value adjustment: tukey method for comparing a family of 4 estimates

Signif. codes: 0 ‘***’ 0.001 ‘**’ 0.01 ‘*’ 0.05 ‘.’ 0.1 ‘ ’ 1
contrast estimate SE df t.ratio p.value
1 - 2 2.052632 1.936114 54 1.060 0.7149
1 - 3 3.368421 1.936114 54 1.740 0.3136
1 - 4 4.368421 1.936114 54 2.256 0.1214
2 - 3 1.315789 1.936114 54 0.680 0.9044
2 - 4 2.315789 1.936114 54 1.196 0.6319
3 - 4 1.000000 1.936114 54 0.516 0.9548

P value adjustment: tukey method for comparing a family of 4 estimates

Signif. codes: 0 ‘***’ 0.001 ‘**’ 0.01 ‘*’ 0.05 ‘.’ 0.1 ‘ ’ 1
contrast estimate SE df t.ratio p.value
1 - 2 -3.761905 2.429108 60 -1.549 0.4155
1 - 3 -4.761905 2.429108 60 -1.960 0.2145
1 - 4 8.238095 2.429108 60 3.391 0.0066
2 - 3 -1.000000 2.429108 60 -0.412 0.9763
2 - 4 12.000000 2.429108 60 4.940 <.0001
3 - 4 13.000000 2.429108 60 5.352 <.0001

P value adjustment: tukey method for comparing a family of 4 estimates

Signif. codes: 0 ‘***’ 0.001 ‘**’ 0.01 ‘*’ 0.05 ‘.’ 0.1 ‘ ’ 1
contrast estimate SE df t.ratio p.value
1 - 2 -3.2380952 2.486221 60 -1.302 0.5650
1 - 3 -2.4285714 2.486221 60 -0.977 0.7631
1 - 4 -4.1428571 2.486221 60 -1.666 0.3503
2 - 3 0.8095238 2.486221 60 0.326 0.9880
2 - 4 -0.9047619 2.486221 60 -0.364 0.9834
3 - 4 -1.7142857 2.486221 60 -0.690 0.9007

P value adjustment: tukey method for comparing a family of 4 estimates

Figure 2B

Active periods PFC

one-way anova
Analysis of Variance of Aligned Rank Transformed Data

Table Type: Repeated Measures Analysis of Variance Table (Type I)
Model: Repeated Measures (aov)
Response: art(variable)

Error Df Df.res F value Pr(>F)
1 time anm:t 3 48 52.546 3.3847e-15 ***

Signif. codes: 0 ‘***’ 0.001 ‘**’ 0.01 ‘*’ 0.05 ‘.’ 0.1 ‘ ’ 1
contrast estimate SE df t.ratio p.value
1 - 2 10.41176 2.962703 48 3.514 0.0052
1 - 3 21.29412 2.962703 48 7.187 <.0001
1 - 4 35.47059 2.962703 48 11.972 <.0001
2 - 3 10.88235 2.962703 48 3.673 0.0033
2 - 4 25.05882 2.962703 48 8.458 <.0001
3 - 4 14.17647 2.962703 48 4.785 0.0001

P value adjustment: tukey method for comparing a family of 4 estimates

Figure 2B

Active periods HP

one-way anova
Analysis of Variance of Aligned Rank Transformed Data

Table Type: Repeated Measures Analysis of Variance Table (Type I)
Model: Repeated Measures (aov)
Response: art(variable)

Error Df Df.res F value Pr(>F)
1 time anm:t 3 48 59.575 3.2938e-16 ***

Signif. codes: 0 ‘***’ 0.001 ‘**’ 0.01 ‘*’ 0.05 ‘.’ 0.1 ‘ ’ 1
contrast estimate SE df t.ratio p.value
1 - 2 11.235294 2.538526 48 4.426 0.0003
1 - 3 23.382353 2.538526 48 9.211 <.0001
1 - 4 31.617647 2.538526 48 12.455 <.0001
2 - 3 12.147059 2.538526 48 4.785 0.0001
2 - 4 20.382353 2.538526 48 8.029 <.0001
3 - 4 8.235294 2.538526 48 3.244 0.0112

P value adjustment: tukey method for comparing a family of 4 estimate

Figure 2D

Power full signal PFC

two-way anova

Figure 2D

Power full signal HP

two-way anova

Analysis of Variance of Aligned Rank Transformed Data	Analysis of Variance of Aligned Rank Transformed Data
Table Type: Repeated Measures Analysis of Variance Table (Type I)<br>Model: Repeated Measures (aov)<br>Response: art(variable)	Table Type: Repeated Measures Analysis of Variance Table (Type I)<br>Model: Repeated Measures (aov)<br>Response: art(variable)
Error Df Df.res F value Pr(>F)<br>1 cond anm:c 1 16 34.631 2.3047e-05 ***<br>2 cond:freq anm:: 3 48 13.074 2.2956e-06 ***<br>---<br>Signif. codes: 0 '***' 0.001 '**' 0.01 '*' 0.05 '.' 0.1 ' ' 1	Error Df Df.res F value Pr(>F)<br>1 cond anm:c 1 16 21.758 0.00025899 ***<br>2 cond:freq anm:: 3 48 17.300 9.4437e-08 ***<br>---<br>Signif. codes: 0 '***' 0.001 '**' 0.01 '*' 0.05 '.' 0.1 ' ' 1
Bonferroni post-hoc comparison<br>delta theta beta gamma<br>0.01538086 0.05145264 0.0033569336 0.0001220703	Bonferroni post-hoc comparison<br>delta theta beta gamma<br>0.31872559 0.04394531 0.0006103516 0.0003051758
**Power active periods PFC**	**Power active periods HP**
two-way anova<br>Analysis of Variance of Aligned Rank Transformed Data	two-way anova<br>Analysis of Variance of Aligned Rank Transformed Data
Table Type: Repeated Measures Analysis of Variance Table (Type I)<br>Model: Repeated Measures (aov)<br>Response: art(variable)	Table Type: Repeated Measures Analysis of Variance Table (Type I)<br>Model: Repeated Measures (aov)<br>Response: art(variable)
Error Df Df.res F value Pr(>F)<br>1 cond anm:c 1 16 19.642 0.00041858 ***<br>2 cond:freq anm:: 3 48 13.957 1.1375e-06 ***<br>---<br>Signif. codes: 0 '***' 0.001 '**' 0.01 '*' 0.05 '.' 0.1 ' ' 1	Error Df Df.res F value Pr(>F)<br>1 cond anm:c 1 16 2.8510 0.1107108<br>2 cond:freq anm:: 3 48 5.6962 0.0020285 **<br>---<br>Signif. codes: 0 '***' 0.001 '**' 0.01 '*' 0.05 '.' 0.1 ' ' 1
Bonferroni post-hoc comparison<br>delta theta beta gamma<br>0.28564453 0.35461426 1.0000000000 1.0000000000	Bonferroni post-hoc comparison<br>delta theta beta gamma<br>0.12207031 1.00000000 1.0000000000 0.4355468750
**Figure 2E**	**Figure 2E**
**Log firing rate full signal PFC**	**Log firing rate full signal HP**
one-way anova<br>Analysis of Variance of Aligned Rank Transformed Data	one-way anova<br>Analysis of Variance of Aligned Rank Transformed Data

Table Type: Repeated Measures Analysis of Variance Table (Type I)
Model: Repeated Measures (aov)
Response: art(variable)

Error Df Df.res F value Pr(>F)
1 time anm:t 3 48 5.0897 0.0038626 **

Signif. codes: 0 '***' 0.001 '**' 0.01 '*' 0.05 '.' 0.1 ' ' 1
contrast estimate SE df t.ratio p.value
1 - 2 2.735294 3.186585 48 0.858 0.8261
1 - 3 10.441176 3.186585 48 3.277 0.0102
1 - 4 9.411765 3.186585 48 2.954 0.0242
2 - 3 7.705882 3.186585 48 2.418 0.0872
2 - 4 6.676471 3.186585 48 2.095 0.1693
3 - 4 -1.029412 3.186585 48 -0.323 0.9882

P value adjustment: tukey method for comparing a family of 4 estimates

Table Type: Repeated Measures Analysis of Variance Table (Type I)
Model: Repeated Measures (aov)
Response: art(variable)

Error Df Df.res F value Pr(>F)
1 time anm:t 3 48 21.092 7.4021e-09 ***

Signif. codes: 0 '***' 0.001 '**' 0.01 '*' 0.05 '.' 0.1 ' ' 1
contrast estimate SE df t.ratio p.value
1 - 2 4.911765 3.228287 48 1.521 0.4329
1 - 3 14.352941 3.228287 48 4.446 0.0003
1 - 4 23.676471 3.228287 48 7.334 <.0001
2 - 3 9.441176 3.228287 48 2.925 0.0261
2 - 4 18.764706 3.228287 48 5.813 <.0001
3 - 4 9.323529 3.228287 48 2.888 0.0286

P value adjustment: tukey method for comparing a family of 4 estimates

Log firing rate active periods PFC

one-way anova
Analysis of Variance of Aligned Rank Transformed Data

Table Type: Repeated Measures Analysis of Variance Table (Type I)
Model: Repeated Measures (aov)
Response: art(variable)

Error Df Df.res F value Pr(>F)
1 time anm:t 3 48 11.305 9.9724e-06 ***

Signif. codes: 0 '***' 0.001 '**' 0.01 '*' 0.05 '.' 0.1 ' ' 1
contrast estimate SE df t.ratio p.value
1 - 2 -4.000000 3.69339 48 -1.083 0.7015
1 - 3 -7.588235 3.69339 48 -2.055 0.1829
1 - 4 -20.294118 3.69339 48 -5.495 <.0001
2 - 3 -3.588235 3.69339 48 -0.972 0.7662
2 - 4 -16.294118 3.69339 48 -4.412 0.0003
3 - 4 -12.705882 3.69339 48 -3.440 0.0064

Log firing rate active periods HP

one-way anova
Analysis of Variance of Aligned Rank Transformed Data

Table Type: Repeated Measures Analysis of Variance Table (Type I)
Model: Repeated Measures (aov)
Response: art(variable)

Error Df Df.res F value Pr(>F)
1 time anm:t 3 48 2.6759 0.057595 .

Signif. codes: 0 '***' 0.001 '**' 0.01 '*' 0.05 '.' 0.1 ' ' 1
contrast estimate SE df t.ratio p.value
1 - 2 -0.8235294 3.42545 48 -0.240 0.9950
1 - 3 -6.4705882 3.42545 48 -1.889 0.2463
1 - 4 -7.8823529 3.42545 48 -2.301 0.1120
2 - 3 -5.6470588 3.42545 48 -1.649 0.3619
2 - 4 -7.0588235 3.42545 48 -2.061 0.1807
3 - 4 -1.4117647 3.42545 48 -0.412 0.9761

P value adjustment: tukey method for comparing a family of 4 estimates

P value adjustment: tukey method for comparing a family of 4 estimates

Figure S1A

Imaginary coherence PFC-HP

two-way anova
Analysis of Variance of Aligned Rank Transformed Data

Table Type: Repeated Measures Analysis of Variance Table (Type I)
Model: Repeated Measures (aov)
Response: art(variable)

Error Df Df.res F value Pr(>F)
1 cond anm:c 1 18 0.274684 0.60660
2 cond:freq anm:: 3 54 0.098025 0.96078

Signif. codes: 0 ‘***’ 0.001 ‘**’ 0.01 ‘*’ 0.05 ‘.’ 0.1 ‘ ’ 1

two-way anova

Bonferroni post-hoc comparison

delta theta beta gamma
1.000000e+00 1.000000e+00 1.000000e+00 1.000000e+00

Figure S1A

Imaginary coherence LEC-OB

two-way anova
Analysis of Variance of Aligned Rank Transformed Data

Table Type: Repeated Measures Analysis of Variance Table (Type I)
Model: Repeated Measures (aov)
Response: art(variable)

Error Df Df.res F value Pr(>F)
1 cond anm:c 1 20 0.55076 0.466636
2 cond:freq anm:: 3 60 2.20480 0.096781 .

Signif. codes: 0 ‘***’ 0.001 ‘**’ 0.01 ‘*’ 0.05 ‘.’ 0.1 ‘ ’ 1

two-way anova

Bonferroni post-hoc comparison

delta theta beta gamma
0.81166840 1.000000e+00 1.000000e+00 0.9159431458

Figure S1C

PPC PFC

two-way anova
Analysis of Variance of Aligned Rank Transformed Data

Table Type: Repeated Measures Analysis of Variance Table (Type I)
Model: Repeated Measures (aov)
Response: art(variable)

Error Df Df.res F value Pr(>F)
1 cond anm:c 1 18 2.5610 0.126930

2 cond:freq anm:: 3 54 2.3506 0.082532 .

Signif. codes: 0 ‘***’ 0.001 ‘**’ 0.01 ‘*’ 0.05 ‘.’ 0.1 ‘ ’ 1

Bonferroni post-hoc comparison

delta theta beta gamma

Figure S1C

PPC HP

two-way anova
Analysis of Variance of Aligned Rank Transformed Data

Table Type: Repeated Measures Analysis of Variance Table (Type I)
Model: Repeated Measures (aov)
Response: art(variable)

Error Df Df.res F value Pr(>F)
1 cond anm:c 1 18 0.76678 0.39275

2 cond:freq anm:: 3 54 1.11065 0.35286

Signif. codes: 0 ‘***’ 0.001 ‘**’ 0.01 ‘*’ 0.05 ‘.’ 0.1 ‘ ’ 1

Bonferroni post-hoc comparison

delta theta beta gamma

Figure S1C

PPC LEC

two-way anova
Analysis of Variance of Aligned Rank Transformed Data

Table Type: Repeated Measures Analysis of Variance Table (Type I)
Model: Repeated Measures (aov)
Response: art(variable)

Error Df Df.res F value Pr(>F)
1 cond anm:c 1 20 2.2892 0.145922

2 cond:freq anm:: 3 60 2.8296 0.045918 *

Signif. codes: 0 ‘***’ 0.001 ‘**’ 0.01 ‘*’ 0.05 ‘.’ 0.1 ‘ ’ 1

Bonferroni post-hoc comparison

delta theta beta gamma

Figure S1C

PPC OB

two-way anova
Analysis of Variance of Aligned Rank Transformed Data

Table Type: Repeated Measures Analysis of Variance Table (Type I)
Model: Repeated Measures (aov)
Response: art(ppcOB)

Error Df Df.res F value Pr(>F)
1 cond_ppcOB an_OB:_OB 1 11 1.70329 0.2185
2 cond_ppcOB:freq_ppcOB a_OB:_OB: 3 33 0.49268 0.6898

Signif. codes: 0 ‘***’ 0.001 ‘**’ 0.01 ‘*’ 0.05 ‘.’ 0.1 ‘ ’ 1

Bonferroni post-hoc comparison

delta theta beta gamma

1.000000e+00 6.751709e-01
1.000000e+00 1.000000e+00

Figure S1D

1/f slope PFC
one-way anova
Analysis of Variance of Aligned Rank Transformed Data

Table Type: Repeated Measures Analysis of Variance Table (Type I)
Model: Repeated Measures (aov)
Response: art(variable)

Error Df Df.res F value Pr(>F)
1 time anm:t 3 54 0.18187 0.90826

Signif. codes: 0 '***' 0.001 '**' 0.01 '*' 0.05 '.' 0.1 ' ' 1
contrast estimate SE df t.ratio p.value
1 - 2 -3.0000000 7.083249 54 -0.424 0.9742
1 - 3 -5.1578947 7.083249 54 -0.728 0.8854
1 - 4 -3.3157895 7.083249 54 -0.468 0.9657
2 - 3 -2.1578947 7.083249 54 -0.305 0.9901
2 - 4 -0.3157895 7.083249 54 -0.045 1.0000
3 - 4 1.8421053 7.083249 54 0.260 0.9938

P value adjustment: tukey method for comparing a family of 4 estimates

Figure S1F

Sample entropy PFC
two-way anova
Analysis of Variance of Aligned Rank Transformed Data

Table Type: Repeated Measures Analysis of Variance Table (Type I)
Model: Repeated Measures (aov)
Response: art(variable)

Error Df Df.res F value Pr(>F)
1 cond anm:c 1 18 0.043354 0.83740
2 cond:freq anm:: 3 54 0.721699 0.54341

1.000000e+00 2.408295e-01
1.000000e+00 1.000000e+00

Figure S1D

1/f slope HP
one-way anova
Analysis of Variance of Aligned Rank Transformed Data

Table Type: Repeated Measures Analysis of Variance Table (Type I)
Model: Repeated Measures (aov)
Response: art(variable)

Error Df Df.res F value Pr(>F)
1 time anm:t 3 54 0.59431 0.62144

Signif. codes: 0 '***' 0.001 '**' 0.01 '*' 0.05 '.' 0.1 ' ' 1
contrast estimate SE df t.ratio p.value
1 - 2 3.684211 7.330003 54 0.503 0.9581
1 - 3 9.263158 7.330003 54 1.264 0.5897
1 - 4 1.894737 7.330003 54 0.258 0.9939
2 - 3 5.578947 7.330003 54 0.761 0.8715
2 - 4 -1.789474 7.330003 54 -0.244 0.9948
3 - 4 -7.368421 7.330003 54 -1.005 0.7470

P value adjustment: tukey method for comparing a family of 4 estimates

Figure S1F

Sample entropy HP
two-way anova
Analysis of Variance of Aligned Rank Transformed Data

Table Type: Repeated Measures Analysis of Variance Table (Type I)
Model: Repeated Measures (aov)
Response: art(variable)

Error Df Df.res F value Pr(>F)
1 cond anm:c 1 18 0.051911 0.82234
2 cond:freq anm:: 3 54 1.105389 0.35498

0.23802948 1.000000e+00
1.000000e+00 1.0000000000

Figure S1D

1/f slope LEC
one-way anova
Analysis of Variance of Aligned Rank Transformed Data

Table Type: Repeated Measures Analysis of Variance Table (Type I)
Model: Repeated Measures (aov)
Response: art(variable)

Error Df Df.res F value Pr(>F)
1 time anm:t 3 60 0.2308 0.87458

Signif. codes: 0 '***' 0.001 '**' 0.01 '*' 0.05 '.' 0.1 ' ' 1
contrast estimate SE df t.ratio p.value
1 - 2 3.8095238 6.694838 60 0.569 0.9409
1 - 3 -1.3333333 6.694838 60 -0.199 0.9972
1 - 4 -0.4761905 6.694838 60 -0.071 0.9999
2 - 3 -5.1428571 6.694838 60 -0.768 0.8685
2 - 4 -4.2857143 6.694838 60 -0.640 0.9186
3 - 4 0.8571429 6.694838 60 0.128 0.9992

P value adjustment: tukey method for comparing a family of 4 estimates

Figure S1F

Sample entropy LEC
two-way anova
Analysis of Variance of Aligned Rank Transformed Data

Table Type: Repeated Measures Analysis of Variance Table (Type I)
Model: Repeated Measures (aov)
Response: art(variable)

Error Df Df.res F value Pr(>F)
1 cond anm:c 1 20 0.4563 0.50709
2 cond:freq anm:: 3 60 1.4887 0.22671

1.00000000 1.000000e+00
1.000000e+00 0.1367187500

Figure S1D

1/f slope OB
one-way anova
Analysis of Variance of Aligned Rank Transformed Data

Table Type: Repeated Measures Analysis of Variance Table (Type I)
Model: Repeated Measures (aov)
Response: art(variable)

Error Df Df.res F value Pr(>F)
1 time anm:t 3 60 0.3423 0.79481

Signif. codes: 0 '***' 0.001 '**' 0.01 '*' 0.05 '.' 0.1 ' ' 1
contrast estimate SE df t.ratio p.value
1 - 2 -0.7142857 6.768396 60 -0.106 0.9996
1 - 3 -3.4761905 6.768396 60 -0.514 0.9555
1 - 4 3.3333333 6.768396 60 0.492 0.9605
2 - 3 -2.7619048 6.768396 60 -0.408 0.9768
2 - 4 4.0476190 6.768396 60 0.598 0.9323
3 - 4 6.8095238 6.768396 60 1.006 0.7465

P value adjustment: tukey method for comparing a family of 4 estimates

Figure S1F

entropy OB
two-way anova
Analysis of Variance of Aligned Rank Transformed Data

Table Type: Repeated Measures Analysis of Variance Table (Type I)
Model: Repeated Measures (aov)
Response: art(variable)

Error Df Df.res F value Pr(>F)
1 cond anm:c 1 20 0.092223 0.76451
2 cond:freq anm:: 3 60 0.134148 0.93933

Signif. codes: 0 '***' 0.001 '**' 0.01 '*' 0.05 '.' 0.1 ' ' 1

Bonferroni post-hoc comparison
delta theta beta gamma
1.000000e+00 1.000000e+00
1.000000e+00 1.000000e+00

Signif. codes: 0 '***' 0.001 '**' 0.01 '*' 0.05 '.' 0.1 ' ' 1

Bonferroni post-hoc comparison
delta theta beta gamma
1.000000e+00 5.787506e-01
1.000000e+00 1.000000e+00

Signif. codes: 0 '***' 0.001 '**' 0.01 '*' 0.05 '.' 0.1 ' ' 1

Bonferroni post-hoc comparison
delta theta beta gamma
1.00000000 1.000000e+00
1.000000e+00 1.0000000000

Signif. codes: 0 '***' 0.001 '**' 0.01 '*' 0.05 '.' 0.1 ' ' 1

Bonferroni post-hoc comparison
delta theta beta gamma
1.00000000 1.000000e+00
1.000000e+00 1.0000000000

Figure S2C

Power full signal PFC
two-way anova
Analysis of Variance of Aligned Rank Transformed Data

Table Type: Repeated Measures Analysis of Variance Table (Type I)
Model: Repeated Measures (aov)
Response: art(variable)

```
          Error Df Df.res F value
Pr(>F)
1 cond      anm:c  1      9
28.661 0.00046016 ***
2 cond:freq anm::  3     27
12.890 2.0587e-05 ***
---
```

Signif. codes: 0 '***' 0.001 '**' 0.01 '*' 0.05 '.' 0.1 ' ' 1

Bonferroni post-hoc comparison
delta theta beta gamma
0.03710938 0.3222656
0.001953125 0.001953125
